# Reappraising intuitions about consciousness

**DOI:** 10.3389/fpsyg.2026.1747924

**Published:** 2026-03-20

**Authors:** Cheongil Kim, Sang Chul Chong

**Affiliations:** 1 Graduate Program in Cognitive Science, Yonsei University, Seoul, Republic of Korea; 2 Department of Psychology, Yonsei University, Seoul, Republic of Korea

**Keywords:** consciousness, intuition about consciousness, metacognitive model, partial awareness, relationship between consciousness and functions

## Abstract

Scientific studies of consciousness derive their legitimacy from individuals’ intuitions about the degree to which measured functions can be associated with consciousness. However, intuitions about consciousness are often regarded as inherently inaccurate and unreliable, as evidenced by cases that appear to reveal counterintuitive dissociations between consciousness and functions. Recent findings challenge this traditional interpretation, suggesting that such cases can be explained by partial awareness that aligns with our intuitions. In this article, we integrate these findings and argue that intuitions about consciousness are more accurate and reliable than previously assumed. Furthermore, by adopting the perspective that consciousness is a metacognitive model of internal representations, we propose that intuitions about consciousness are metacognitive models of the relationship between internal representations and functions. This functional reframing not only explains why intuitions about consciousness can be accurate and reliable but also facilitates systematic investigations by linking them to well-studied cognitive functions, such as confidence, target templates, attention modes, and awareness of absence.

## 1 Introduction

### 1.1 Intuitions about consciousness

**Figure 1:**
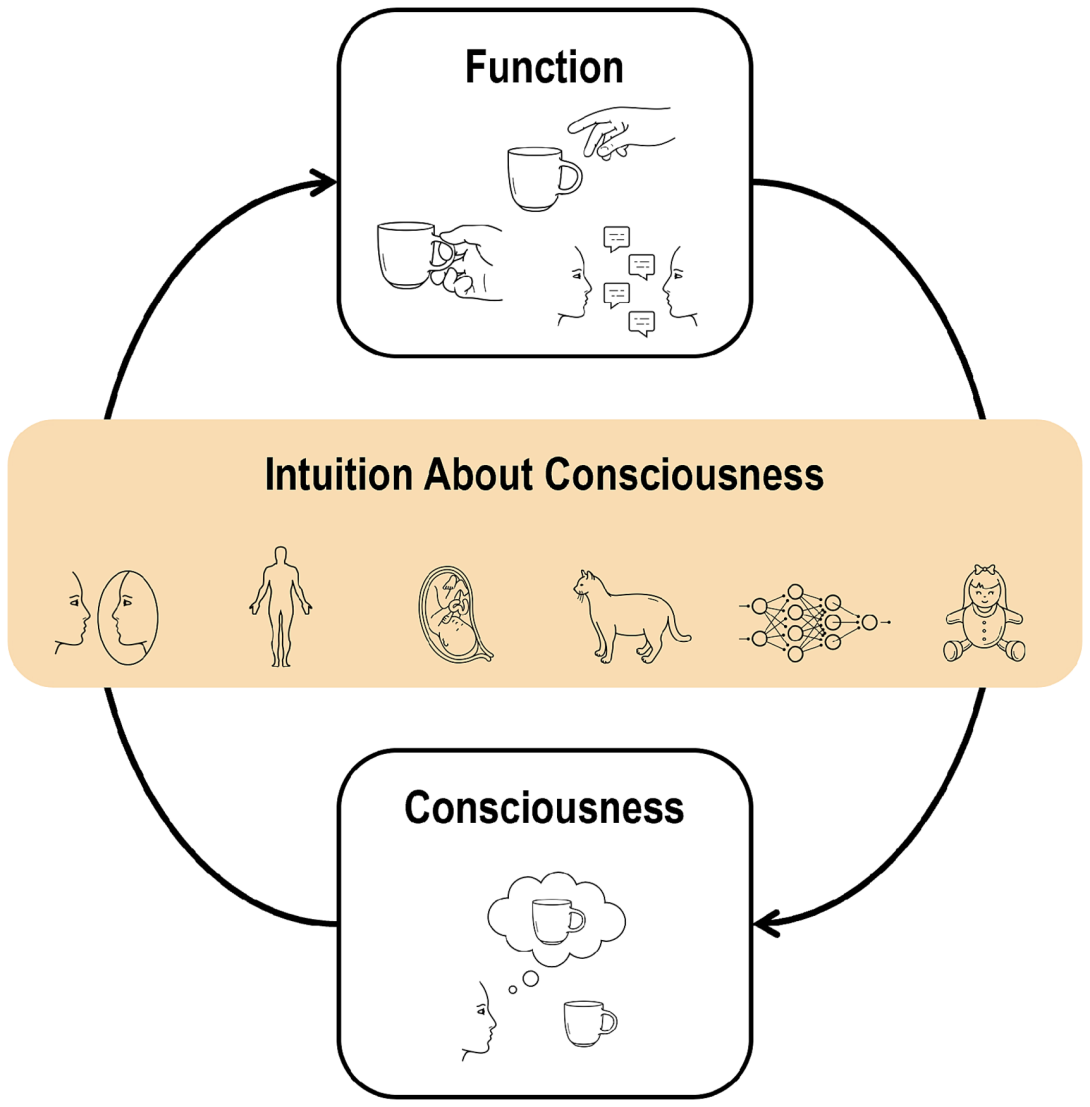
Intuitions about consciousness allow us to predict possible functions based on conscious experiences and infer conscious experiences from observed functions. These intuitions can extend beyond the self, enabling us to attribute consciousness to other people, nonhuman animals, artificial intelligences, and even objects and interact with them accordingly.

Throughout our lives, we develop an understanding of the relationship between our conscious experiences and functions, such as behaviors, verbal reports, and cognitive processes. This lifelong learning shapes our intuitions about consciousness, enabling us to predict possible functions based on conscious experiences and infer conscious experiences from observed functions (Figure 1). For example, imagine that you are consciously perceiving a cup in front of you. You might believe that you can reach out and grasp it properly or describe its color and shape in detail. If you can perform these functions, you are likely to conclude that you are consciously perceiving the cup.

These intuitions about consciousness often extend beyond the self, enabling attributions of consciousness to other people, nonhuman animals, artificial intelligences, and even objects—a phenomenon commonly referred to as theory of mind, mind perception, or anthropomorphism (Colombatto and Fleming, 2024; Epley et al., 2007; Frith and Frith, 2005; Goldman, 2006; Gray et al., 2007; Knobe and Prinz, 2008; Leslie et al., 2004; Waytz et al., 2010). Based on observable functions, we automatically infer others’ conscious experiences and interact with them accordingly. Intuitions about consciousness are referred to as such because they resemble gut feelings rather than structured knowledge. Specifically, they are immediate in that they are not preceded by any conscious process of reflective thinking (Evans, 2010; Pearson, 2024; Thompson, 2014). In other words, intuitions themselves are conscious in that they manifest as an experiential character (i.e., feelings), whereas their underlying sources remain unconscious (“knowing without knowing how”). For example, although we explicitly know that artificial intelligences (e.g., chatbots) lack consciousness, we can still feel for and treat them as if they are conscious. Similarly, some argue for the logical possibility of a philosophical zombie—a being physically and behaviorally indistinguishable from humans yet lacking conscious experience (Chalmers, 1996)—but they may nonetheless feel for and treat their families and friends as if they are conscious.

Since previous studies have used the term intuitions about consciousness in various ways (Colombatto and Fleming, 2024; Fischer and Sytsma, 2021; Knobe and Prinz, 2008), this article seeks to clarify its scope and to discuss intuition about consciousness within the following boundaries. First, in this article, intuitions about consciousness refer to implicit and automatic inferences rather than explicit and structured knowledge. Second, they encompass both consciousness-to-function (i.e., predicting possible functions based on consciousness) and function-to-consciousness directions (i.e., attributing consciousness to observed functions), whereas previous research has focused on the latter. Lastly, our discussion centers on intuitions about the relationship between one’s own consciousness and functions, in contrast to previous research that has investigated how such intuitions extend to other agents.

### 1.2 The importance of intuitions about consciousness in scientific studies of consciousness

**Figure 2:**
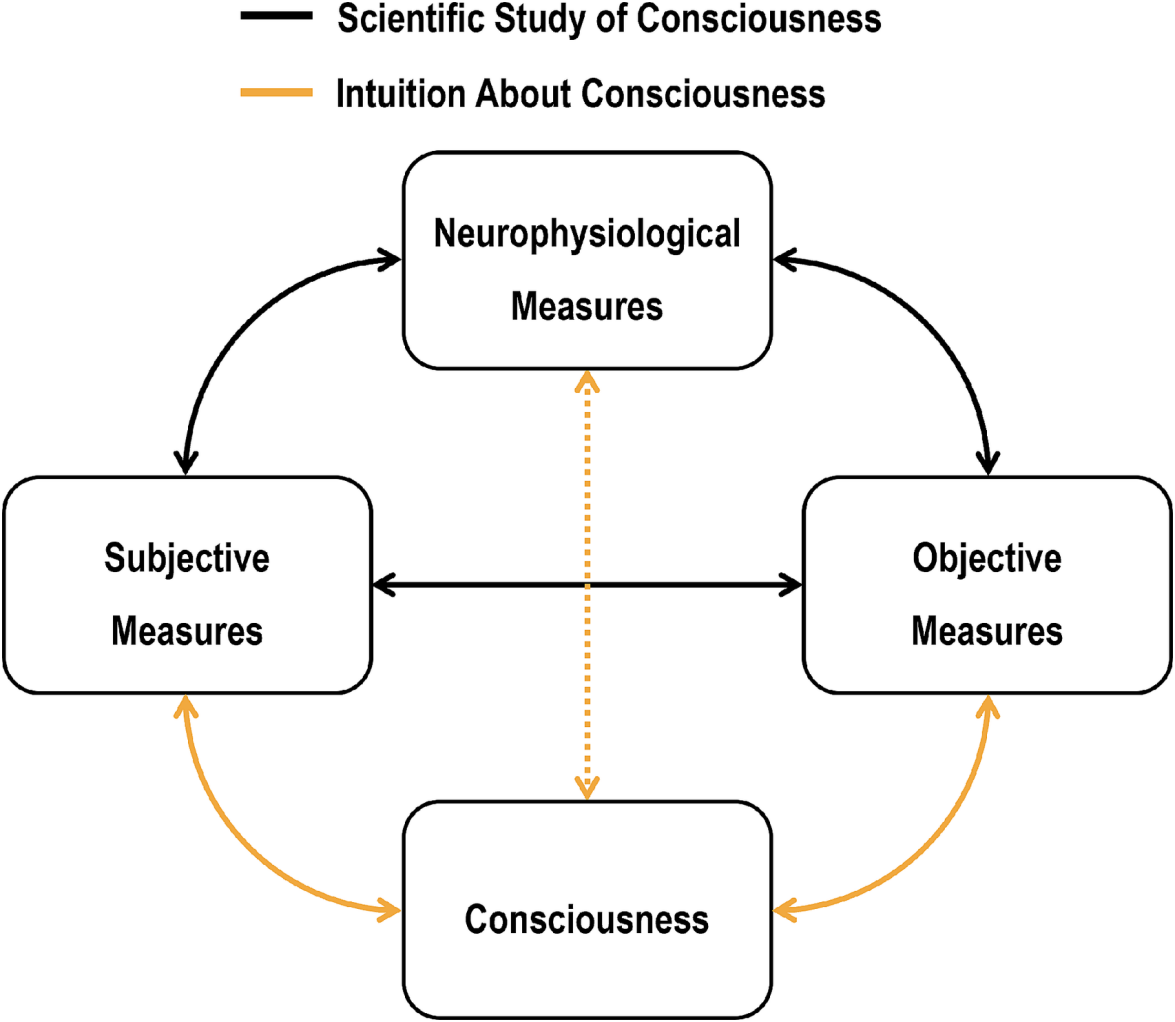
Intuitions about consciousness and scientific studies of consciousness. Scientific studies of consciousness aim to create scientific models of the relationship between measurable functions, such as behavioral (e.g., subjective or objective measures) and neurophysiological measures. Intuitions about consciousness are internal models of the relationship between consciousness and these functions. The relationship between consciousness and neurophysiological measures is represented by a dotted line, indicating a weak intuition about the relationship with the possibility of learning it further, for example, through neurofeedback.

Scientific studies of consciousness rely on individuals’ intuitions about consciousness. Consciousness is defined as a subjective or first-person phenomenon, which means it is inherently inaccessible to third-person examination (Chalmers, 1995; Nagel, 1974). Consequently, researchers rely on observable functions—such as self-reports, behaviors, and neurophysiological measures—that are presumed to be closely associated with consciousness (Bayne et al., 2024; Blake, 2022; Carrasco and Spering, 2024; He, 2023; Jimenez et al., 2025; Kiefer et al., 2023; Kim and Blake, 2005; Overgaard, 2017; Overgaard and Kirkeby-Hinrup, 2021; Seth et al., 2008; Weiskrantz, 1997). For example, observers’ self-reports of conscious experiences (subjective measures) or above-chance discrimination performance (objective measures) have traditionally been regarded as primary measures of consciousness (Kiefer et al., 2023; Overgaard and Sandberg, 2021). By investigating the relationship between these measures and other behavioral or neurophysiological measures (Cogitate Consortium, 2025; Melloni et al., 2021; Sergent et al., 2021; Seth et al., 2008), scientific studies of consciousness have extended our knowledge about the relationship between functions associated with consciousness (Figure 2).

Given the above, whether certain scientific knowledge pertains to consciousness depends on researchers’ or readers’ intuitions about the association between measured functions and consciousness. In other words, scientific studies of consciousness derive their legitimacy from individuals’ intuitions about consciousness. Although nonintuitive measures of consciousness, such as neurophysiological measures with no-report paradigms, could potentially replace intuitive measures (Brascamp et al., 2015; Hatamimajoumerd et al., 2022; Owen et al., 2006; Sergent et al., 2021; Tsuchiya et al., 2015); but see Block (2019), Carrasco and Spering (2024), and Duman et al. (2022), their validity should be evaluated based on how closely they are associated with intuitive measures of consciousness.

Therefore, systematic investigations into intuitions about consciousness are crucial for advancing scientific studies of consciousness. For example, empirical research should address key questions: Do observers believe that self-reports reflect their consciousness more accurately or exhaustively than other behavioral measures? If so, which type of self-reports (e.g., visibility ratings, confidence ratings, or the perceptual awareness scale) is regarded as the most reliable? And if no existing measures sufficiently align with observers’ intuitions about consciousness, what kind of measure could? Investigating these questions will deepen our understanding of how to scientifically assess and interpret consciousness.

In sum, the present study underscores the importance of developing an operational definition of consciousness that accommodates individuals’ intuitions about the relationship between consciousness and function. Accordingly, rather than presupposing the existence of functionally irrelevant and empirically inaccessible forms of consciousness, we adopt an optimistic stance that treats consciousness as closely linked to measurable functions. For this reason, the notion of consciousness used throughout the manuscript is primarily restricted to functionally relevant and accessible aspects of consciousness. In addition, we focus on research concerning the contents of visual awareness (i.e., visual representations), where this approach has been mostly extensively developed.

Importantly, this approach does not enable direct tests of claims concerning forms of phenomenal consciousness that are entirely independent of function. Nevertheless, it provides a principled basis for critically re-evaluating influential studies that argue for dissociations between consciousness and function while simultaneously relying on functional measures as putative indicators of consciousness. Such studies often highlight empirical dissociations among measures that are commonly taken to reflect consciousness (e.g., subjective reports and objective performance), but some interpretations of these findings may be overstated. Our aim is therefore to delimit the boundary within which dissociations between consciousness and function can be plausibly asserted, thereby contributing to reducing skepticism about the scientific viability of consciousness research.

## 2 Arguments for counterintuitive dissociation between consciousness and functions

Despite their importance, systematic investigations of intuitions about consciousness are lacking. This scarcity may stem from the belief that intuitions about consciousness, or introspection itself, are inherently inaccurate and unreliable (Goldman, 2006; Peters, 2025; Schwitzgebel, 2008). Indeed, a large body of scientific and philosophical studies of consciousness has focused on striking cases that appear to demonstrate the inaccuracy and unreliability of our intuitions of consciousness. In particular, these studies have suggested that our intuitions overestimate conscious functions, or underestimate unconscious functions, based on cases demonstrating counterintuitive dissociation between consciousness and functions.

### 2.1 Functions without consciousness

One of the most influential demonstrations of dissociation between consciousness and functions is blindsight, a neuropsychological condition defined by residual visual functions—such as voluntary discrimination of orientations, shapes, directions of motion, or colors—despite the absence of visual awareness following damage to the primary visual cortex (de Gelder et al., 2008; Pöppel et al., 1973; Weiskrantz et al., 1974). Blindsight challenges the intuition that such voluntary functions require consciousness, serving as strong evidence for the counterintuitive dissociation between consciousness and functions.

This striking dissociation between consciousness and functions has significantly influenced theories of consciousness. Despite controversy over the underlying mechanisms of consciousness, many theories of consciousness commonly assume that consciousness and functions can be dissociated [see Cohen and Dennett (2011) for a review]. For example, higher-order theories propose that re-representations of first-order representation (i.e., early sensory processing) are necessary for consciousness, while first-order representations alone contribute to functions but not to consciousness (Brown et al., 2019; Lau and Rosenthal, 2011).

To support this view, researchers have attempted to replicate findings from blindsight-like phenomena in healthy individuals to demonstrate functions without consciousness, or unconscious perception (Brown et al., 2019; Michel, 2023b; Peters et al., 2017). Furthermore, they have emphasized the importance of comparing consciousness across conditions with matched functions when investigating neural correlates of consciousness to eliminate confounding effects from unconscious processing (relative blindsight; Lau, 2022; Lau and Passingham, 2006; Rahnev et al., 2011). For example, Lau and Passingham (2006) examined a relative blindsight case in which subjective reports of consciousness were lower for short time intervals than for long time intervals between target and mask onset in metacontrast masking, while objective discrimination performance was matched. Thus, unconscious perception and relative blindsight in healthy individuals challenge the intuition that voluntary functions go hand in hand with consciousness.

### 2.2 Consciousness without functions

In contrast to the above discussion, first-order theories suggest that first-order representations alone are sufficient for consciousness and that higher-order representations contribute only to post-perceptual functions, not to consciousness itself (e.g., local theories of consciousness; Block, 2011; Lamme, 2010). Proponents of first-order theories point to cases of consciousness without accompanying functions, where observers report rich conscious experiences but fail to report specific contents of those experiences (Bronfman et al., 2014; Sligte et al., 2008; Tsuchiya et al., 2015), such as inattentional blindness and change blindness. The former refers to the failure to notice an unexpected yet salient stimulus (e.g., the “invisible gorilla”; Simons and Chabris, 1999) when one’s attention is focused on a demanding task (Mack and Rock, 1998), while the latter refers to the failure to detect a significant change between images (Rensink et al., 1997; Simons and Levin, 1997).

First-order theories suggest that inattentional blindness and change blindness arise from failures not in conscious perception but in post-perceptual processing. For example, inattentional blindness may result from a failure to remember a task-irrelevant target (Chen and Wyble, 2015; Wolfe, 1999). Additionally, change blindness may stem from a failure to retain the pre-change image (Bronfman et al., 2014; Lamme, 2003) or compare it with the post-change image (Mitroff et al., 2004). In this regard, such theories propose that consciousness overflows what can be cognitively accessed and functionally available. This overflow explanation, which has been applied to striking cases of failures to report where failures in post-perceptual processing are unlikely (Kim and Chong, 2024b; Simons and Chabris, 1999; Ward and Scholl, 2015), challenges the intuition that consciousness of a target (e.g., the “invisible gorilla”) is accompanied by at least basic functions, such as the ability to report the presence of the target (e.g., reporting the presence of the gorilla).

## 3 Partial awareness: an intuitive account of seemingly counterintuitive dissociation

While an increasing number of studies have adopted the dissociation between consciousness and functions as a commonly accepted standpoint, some researchers argue that the traditional interpretation of these counterintuitive findings may be flawed (Cova et al., 2021; Kim and Chong, 2021, 2022, 2024a, 2024b; Kouider et al., 2010; Phillips, 2021). This camp proposes that such findings do not reflect a dissociation between consciousness and functions but rather a mismatch between the measures used to assess them.

The core assumption here is that consciousness is not an all-or-none but rather a graded phenomenon (Kim and Chong, 2021; Kouider et al., 2010; Overgaard and Sandberg, 2021). Supporting this view, previous studies have demonstrated that observers report partial and graded awareness of individual items when their awareness is degraded through various techniques, such as brief stimulus presentation, masking, attentional blink, or continuous flash suppression (Andersen et al., 2016; Cohen et al., 2023; Elliott et al., 2016; Hong and Blake, 2009; Jimenez et al., 2020; Kim and Chong, 2021, 2024a, 2024b; Kobylka et al., 2017; Magazzini et al., 2016; Overgaard et al., 2006).

The partial awareness hypothesis posits that representational contents of an individual item can be subdivided and that observers can access these contents independently. For example, in hierarchical processing, observers can access low-level representational contents (e.g., colors, textures, or forms) but not higher-level ones (e.g., categories or identities; Elliott et al., 2016; Jimenez et al., 2020; Kobylka et al., 2017; Magazzini et al., 2016). Similarly, in parallel processing, observers can access only some representational contents, such as colors but not forms (Hong and Blake, 2009) or low spatial frequencies but not high spatial frequencies (Kim and Chong, 2021, 2024a, 2024b). This empirical evidence for partial and graded awareness emphasizes the importance of using refined measures of consciousness to narrow the gap between experienced and reported consciousness when studying the relationship between consciousness and functions. However, the possibility of partial and graded awareness has not been sufficiently considered in previous studies.

It is worth noting that the partial awareness hypothesis is not intended to deny the possibility of dissociations between consciousness and function. Rather, it aims to clarify the relationship among different measures, thereby delimiting the boundary within which dissociations between consciousness and function can be plausibly asserted. Specifically, we suggest that the extent of such dissociations may have been overestimated in studies that did not adequately account for partial awareness.^1^

### 3.1 Functions without consciousness

Some researchers have recently suggested that blindsight may represent degraded conscious vision accompanied by conservative response biases rather than complete unconsciousness (Overgaard and Sandberg, 2021; Phillips, 2021). In this view, residual conscious vision contributes to above-chance performance in forced-choice tasks but is too weak to be confidently reported as conscious vision. Supporting this interpretation, studies have shown that blindsight seems to disappear when graded measures, such as four-level scales (e.g., perceptual awareness scale: no experience, weak experience, almost clear experience, and clear experience; Ramsøy and Overgaard, 2004), are utilized. Residual voluntary functions in blindsight may, thus, correspond to weak but conscious vision (Mazzi et al., 2016; Overgaard et al., 2008).

Similarly, several studies have found that previously reported cases of unconscious perception fail to replicate when using refined measures that account for partial awareness. These findings suggest that previous findings of unconscious perception can be explained by residual partial awareness that went unreported due to the use of dichotomous response options and conservative response biases (e.g., seen/not seen; Balsdon and Clifford, 2018; Peters and Lau, 2015; Phillips, 2016; Rajananda et al., 2020). Like blindsight cases, studies have shown that unconscious perception seems to disappear when graded measures are applied (Biderman and Mudrik, 2018; Gelbard-Sagiv et al., 2016; Lohse and Overgaard, 2019). However, it is worth noting that we are not denying the possibility of blindsight and unconscious perception (Michel, 2023b). Rather, we argue that their extent may have been overestimated in studies that did not adequately account for partial awareness.

Partial awareness also provides an explanation for relative blindsight by suggesting that the contents of consciousness can be subdivided as either task-relevant or task-irrelevant (Kahneman, 1968; Michel, 2023b; Newell and Shanks, 2014). Subjective measures, such as visibility or confidence ratings, are more permissively influenced by various contents and contextual factors than objective task performance (Abid, 2019; Firestone and Scholl, 2016; Kim and Chong, 2022; Michel, 2023b). Accordingly, it is possible that, in relative blindsight cases, objective task performance and subjective reports may rely on different contents, which may explain the apparent dissociation. For example, while task-relevant contents (e.g., target orientation) are controlled to be the same, task-irrelevant contents (e.g., time intervals between target and mask onset) that influence subjective reports (e.g., confidence ratings of orientation discrimination) can vary across conditions (Kim and Chong, 2022; Rausch et al., 2018; Shekhar and Rahnev, 2021). Additionally, different stimulus manipulations (e.g., size, duration, noise contrast, and orientation) can produce different effects on objective task performance and subjective reports (Fung et al., 2025). This suggests that relative blindsight may reflect the alignment of performance for a specific function through different combinations of representational contents rather than providing a contrast between conscious and unconscious processing of a specific content.

### 3.2 Consciousness without functions

Partial awareness provides an intuitive explanation for the seemingly counterintuitive dissociation between rich consciousness and impoverished functions. Traditionally, beliefs in rich consciousness have been inferred from anecdotal reports by observers or researchers. However, recent studies with refined measures of consciousness have demonstrated that observers do not believe they have as rich and detailed consciousness as previously assumed. For example, Cova et al. (2021) revealed that, when given options for partial awareness, observers in Sperling’s and Landman’s experiments reported partial and generic visual experiences when viewing multiple letters and rectangles simultaneously. Additionally, Kim and Chong (2024a) demonstrated that observers are aware of decreases in perceptual resolution with eccentricity through in-the-moment metacognition during tasks, challenging the assumption that observers believe uniformly rich and detailed visual experiences across the visual field. Overall, these findings suggest that observers’ consciousness may not be as rich as previously assumed and could correspond to their impoverished functions.

Moreover, recent studies suggest that observers’ functions may not be as impoverished as previously assumed (Haun, 2021; Rosenholtz, 2020). For example, Nartker et al. (2024) found that, in inattentional blindness, observers who denied noticing a target were still able to discriminate its location, color, and shape. Furthermore, Kim and Chong (2024b) reported that, when multiple faces were presented simultaneously, observers failed to detect the loss of high but not low spatial frequency information of faces, which suggests partial blindness to individual faces. Similarly, studies on ensemble perception indicate that observers can access and utilize summary statistics of a complex scene, such as the mean and variance of a group of similar items, even when they lack detailed information about each individual item (Baek and Chong, 2020; Cohen et al., 2016; Ward et al., 2016; Whitney and Yamanashi Leib, 2018). Additionally, studies using massive-report or free-report paradigms reveal that observers’ reports on a briefly viewed scene are much richer than previously thought (Chuyin et al., 2024; Qianchen et al., 2022). These findings caution against generalizing the failure of one function to all functions related to individual items or scenes and suggest that observers may have partial functions corresponding to partial awareness.

## 4 The relationship between consciousness and functions

With an appropriate reappraisal, our intuitions about consciousness seem to be more accurate than previously assumed. Please note that we do not claim that our intuitions about consciousness are perfect but rather that they are sufficiently accurate and reliable to be scientifically studied. Additionally, we distinguish between “counterintuitive” and “nonintuitive” cases. While we acknowledge the existence of many nonintuitive relationships between consciousness and functions, it is only counterintuitive cases that demonstrate inaccuracies in our intuitions. Therefore, in this article, we focus on cases where observers experience strong intuitive or counterintuitive impressions.

The accurate intuitions may stem from a reliable correlation between consciousness and functions, as demonstrated by empirical studies contrasting conscious and unconscious functions (Cohen and Dennett, 2011). Traditionally, this reliable correlation has been interpreted to indicate that consciousness enables certain functions or is necessary for them. However, an alternative interpretation is also possible: certain functions enable consciousness or are necessary for it. This perspective aligns with an emerging theoretical view that proposes consciousness as a metacognitive model of internal representations (Cleeremans et al., 2020; Cortese and Kawato, 2024; Fleming and Michel, 2025; Gershman, 2019; Lau, 2022; Webb and Graziano, 2015). According to this view, consciousness results from an implicit assessment (i.e., implicit metacognition) of the strength and reliability of internal representations or is the assessment itself. This assessment seems to be processed implicitly, resulting in a stubborn “assertoric force.” It tells us about what is happening here and now in ways that we cannot ignore or control through explicit beliefs and thoughts (Lau, 2022). In this framework, consciousness provides agents with models, or indicators, of which internal representations are being attended to and stably processed. Thus, consciousness of certain contents arises when the internal representations of those contents are reliable enough to perform certain functions. Moreover, agents rely on consciousness to determine whether they will perform those functions, creating a reliable correlation between consciousness and functions.

## 5 Reframing intuitions about consciousness as metacognitive models of the relationship between internal representations and functions

We propose that intuitions about consciousness mediate the relationship between consciousness and functions, serving as *metacognitive models of the relationship between internal representations and functions*. Intuitions about consciousness may arise from an implicit assessment (i.e., implicit metacognition) of this relationship because individuals typically have access only to inferred internal representations or functions (the products of inference), rather than explicit and structured knowledge of the relationship between them (the inference process itself).

It is worth noting that this functional reframing does not aim to argue that the phenomenology of intuition can be exhaustively reduced to, or replaced by, its functional role. It does not deny the possibility that intuitions may have a phenomenological profile that exceeds their functional role, nor that additional function may need to be incorporated to account for their rich phenomenological profile (e.g., emotional experiences; Loev, 2022). Rather, this functional reframing is intended to provide a useful framework for the scientific study of intuitions about consciousness. Specifically, it offers systematic accounts of when and why intuitions about consciousness arise, why they reliably track consciousness-function relations, and how they relate to other functionally well-defined phenomena (e.g., attention and confidence). Moreover, it supports multi-level operationalization of intuitions about consciousness across psychological, behavioral, neural, and computational measures, thereby contributing to a more comprehensive understanding of intuitions about consciousness.^2^

**Figure 3:**
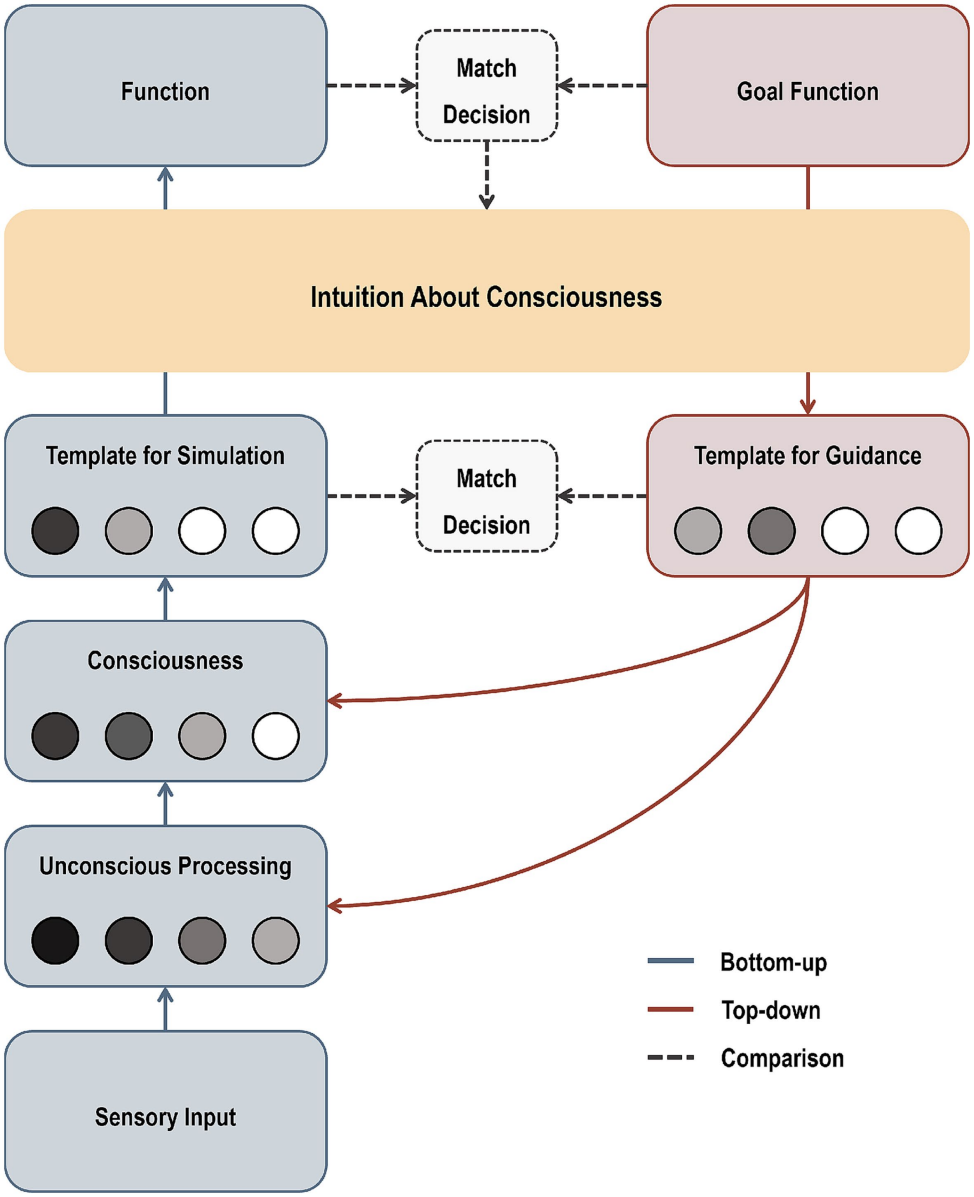
A framework for incorporating intuitions about consciousness into information processing.

Figure 3 illustrates our framework, visualizing how intuitions about consciousness can mediate the relationship between consciousness and functions during information processing. Our framework hypothesizes three main modes of information processing: (1) bottom-up, (2) top-down, and (3) comparison.

### 5.1 Bottom-up processes

Bottom-up sensory inputs (e.g., visual stimuli) are processed through hierarchical (Craik and Lockhart, 1972; Grill-Spector and Malach, 2004; Hubel and Wiesel, 1968) and parallel pathways (Livingstone and Hubel, 1988; Nassi and Callaway, 2009; Zeki et al., 1991), which results in the subdivision of several representational contents (e.g., orientation, spatial frequency, motion, color, form, texture, category, and identity). Consciousness is an metacognitive model of these internal representations, monitoring both the content being processed (content parameter) and its reliability (weight parameter) at any given moment (Fleming and Michel, 2025; Lau, 2022; Webb and Graziano, 2015).^3^ Depending on these parameters, observers can have diverse forms of partial awareness in hierarchical (Kouider et al., 2010) and parallel ways (Kim and Chong, 2021). Some conscious representational contents are selected and weighted to generate a template for simulation and planning (Doll et al., 2012; Fleming and Michel, 2025; Hunt et al., 2021; Lau, 2022; Mattar and Lengyel, 2022; Webb and Graziano, 2015). Based on their intuitions about consciousness, observers predict which functions can be performed with the template for simulation (e.g., whether the representational contents of a cup are sufficient to be able to properly reach out and grasp it or describe its color and shape in detail) and determine whether they will perform those functions.

### 5.2 Top-down processes

When there is a goal function, observers utilize their intuitions about consciousness to predict the required representational contents and their reliability for performing the function (e.g., which representational contents of a cup are necessary to properly reach out and grasp it or describe its color and shape in detail). This generates a template for guidance (e.g., target templates or expectations), which can tune parameters of bottom-up models of internal representations through top-down modulations (de Lange et al., 2018; Duncan and Humphreys, 1989; Kok et al., 2017; Wolfe, 2021; Yu et al., 2023). These top-down modulations include different forms, such as spatial attention, feature-based attention, object-based attention, expectations, and cognitive penetrations, which facilitate the processing of goal-relevant representational contents (Baldauf and Desimone, 2014; Carrasco, 2011; Gilbert and Li, 2013; Harrison and Tong, 2009; Lupyan et al., 2020; Serences and Boynton, 2007; Vetter and Newen, 2014; Vetter et al., 2024; Xu, 2017; Yu et al., 2023).

### 5.3 Comparison processes

By comparing the template for guidance with the template for simulation (match decision), observers determine whether to proceed with the goal function or gather more information (Engel and Wang, 2011; Miller and Desimone, 1994; Thomas et al., 2024; Wolfe, 2021; Xu, 2017; Yu et al., 2023). After performing functions, observers compare the goal and performed functions (Butler et al., 2008; den Ou et al., 2012; Miall and Wolpert, 1996; Ohlsson, 1996; Schultz and Dickinson, 2000; Shadmehr et al., 2010). If errors exist between them, intuitions about consciousness are updated to minimize errors, enabling more accurate and efficient information processing. For example, if the conducted functions fall short of the goal functions, the criteria for the necessary contents and the intensity of consciousness are raised to meet the demands of the goal functions. By contrast, if the conducted functions exceed the goal functions, these criteria are lowered to optimize efficiency in information processing.

In sum, we incorporate intuitions about consciousness into information processing by defining them as metacognitive models of the relationship between consciousness and functions. According to our proposed framework, intuitions about consciousness should be accurate and reliable, as they mediate between consciousness and functions; they determine what functions observers will voluntarily perform with their consciousness and shape consciousness itself to align with goal functions.^4^

## 6 Connecting intuitions about consciousness to well-studied cognitive functions

If intuitions about consciousness can be reframed as metacognitive models of the relationship between internal representations and functions, this means they can be studied in relation to well-studied cognitive functions, such as confidence, target templates, attention modes, and awareness of absence.

### 6.1 Confidence

Confidence refers to a sense of certainty about one’s functions, arising from metacognitive monitoring of internal representations (Fleming, 2024; Rahnev, 2021). Confidence ratings have been widely used to measure how individuals monitor their internal representations (Rahnev et al., 2020). Intuitions about consciousness can be linked to confidence, as they may enable observers to predict functions based on their internal representations. Thus, by adapting studies on how confidence ratings are generated (Fleming, 2024; Mamassian, 2016; Michel, 2023a; Rahnev et al., 2022), we can investigate intuitions about consciousness. Specifically, observers’ intuitions about consciousness can be assessed by examining whether and how the modulation of specific representational contents affects confidence ratings for specific functions. Additionally, the accuracy of these intuitions can be determined by examining how well the confidence ratings of the function track the effect of content modulation on the function (Kim and Chong, 2025).

This approach helps in evaluating whether a specific function is a proper measure of consciousness in terms of intuitions of consciousness.^5^ For example, if modulating a specific representational content (e.g., orientation) improves the performance of a specific function (e.g., orientation discrimination) but has no effect on confidence rating, this suggests an inaccurate model of the relationship between the content and function (i.e., inaccurate intuition about consciousness). Such a case also reveals that the content might implicitly (if included in consciousness but not a template for simulation) or unconsciously (if excluded even from consciousness; Michel, 2023a) influence performance of the function. Consequently, the function may not serve as a proper measure of consciousness for the content in terms of intuitions about consciousness. Conversely, a function can be regarded as a proper measure of consciousness if confidence ratings accurately reflect the effect of content modulation on the function.

### 6.2 Target templates

In models of visual search, target templates (or attentional templates) characterize the representational contents of a target (e.g., “red” and “circular” if a target is an apple) held in working or long-term memory (Duncan and Humphreys, 1989; Wolfe, 2021; Yu et al., 2023). Target templates guide attention to a potential target by facilitating the processing of target-relevant contents (attentional guidance), and they support decisions about whether an attended object matches the target (target-matching decisions). Traditionally, target templates were considered complete sets of targets’ representational contents. However, recent studies suggest that they comprise only a subset of associated contents optimized for visual search in specific task contexts (Olivers et al., 2011; Yu et al., 2023). Furthermore, depending on the goal (e.g., attentional guidance versus target-matching decisions or distinguishing targets from dissimilar versus similar distractors), observers can flexibly determine the resolution of target templates (e.g., coarse versus fine information about the target; Yu et al., 2022a, 2022b).

Intuitions about consciousness may serve as mechanisms that generate target templates optimized for specific goal functions. As models of the relationship between internal representations and functions, intuitions about consciousness enable observers to predict which representational contents are necessary and sufficient for performing goal functions. Therefore, by examining target templates, we can infer intuitions about consciousness. Specifically, if target templates include specific representational contents for specific goal functions, this suggests that observers possess intuitions about the relationship between those contents and functions.

Target templates can be characterized by examining which representational contents benefit from attentional guidance, as evidenced by improved task performance and sensory gains in neural activities (Baldauf and Desimone, 2014; Malcolm and Henderson, 2009; Serences and Boynton, 2007; Yu et al., 2022a, 2022b, 2023). The resolution of target templates can also be inferred by attentional guidance strategies, such as attentional breadth, functional visual field, and search termination. These strategies reflect the expected difficulty of finding a target and individuals’ metacognitive beliefs about the extent to which detailed representational contents are necessary. Easier searches are associated with broader attentional breadth, more items in the functional visual field, and shorter search termination times, indicating coarse target templates. By contrast, more challenging searches are associated with narrower attentional breadth, fewer items in the functional visual field, and longer search termination times, reflecting fine target templates (Goodhew, 2020; Hulleman and Olivers, 2017; Mazor and Fleming, 2022; Wolfe, 2021; Zheng and Chong, 2025).

By adapting these approaches to the study of attentional guidance, we can infer whether observers have intuitions about the relationship between goal functions and representational contents. Additionally, we can assess the accuracy of these intuitions by examining whether attentional guidance enhances the performance of goal functions.

### 6.3 Attention modes: focused attention and ensemble perception

Focused attention and ensemble perception, or distributed attention, have been proposed as two distinct strategies for dealing with the limited capacity of our visual system (Chong and Evans, 2011; Treisman, 2006). Focused attention selectively enhances the processing of important information within a scene (Carrasco, 2011), whereas ensemble perception extracts the gist, or summary statistics, from complex and redundant information (Whitney and Yamanashi Leib, 2018). Baek and Chong (2020) propose that observers can flexibly deploy attention in either mode depending on task demands. Specifically, focused attention is employed for tasks that require detailed information or the integration of multiple features from a single item. This mode of attention aligns with attentional guidance in visual search, as it aims to distinguish a target from distractors using templates for guidance.

By contrast, ensemble perception is used for tasks that require summarizing a scene, such as extracting summary statistics (e.g., mean or variance) of a single feature across multiple items. It encompasses not only low-level features such as orientation (Parkes et al., 2001) and size (Chong and Treisman, 2003) but also high-level features such as emotion (Haberman and Whitney, 2007), gender (Oswald et al., 2023), group diversity (Kardosh et al., 2022; Phillips et al., 2018), and entitativity (Goldenberg et al., 2020), thereby providing the basis for norms of social perception. The ability to modulate attention modes based on task demands is, therefore, crucial for optimal visual performance across a wide range of contexts (Chong and Evans, 2011; Evans and Chong, 2012; Mann and Walker, 2003; Wolfe et al., 2011).

Intuitions about consciousness may serve as mechanisms that modulate attention modes in accordance with goal functions. These intuitions enable observers to generate templates for guidance based on task demands (e.g., whether the task requires processing multiple features of a single item or a single feature across multiple items). As discussed in the Target templates section, we can infer intuitions about consciousness by examining which representational contents benefit from attentional guidance. Additionally, intuitions about consciousness can be assessed by investigating which contents contribute to the accuracy and bias of ensemble representations (Cha and Chong, 2018; Jeong and Chong, 2021; Kim et al., 2025; Lee and Chong, 2024; Sun and Chong, 2020). Specifically, by examining which features or items influence ensemble judgments (e.g., overall mood judgments), we can investigate observers’ intuitions about the relationship between contents and functions.

### 6.4 Awareness of absence

Intuitions about consciousness can also be investigated by studying observers’ decisions on whether internally generated templates for guidance (e.g., target templates, attentional templates, or top-down expectations) match externally generated templates for simulation (match decisions). Specifically, we can assess whether observers include a certain representational content in their templates for guidance by examining whether they are aware of the absence of that content in their templates for simulation (awareness of absence; Mazor and Fleming, 2020). If the templates for guidance include the content, observers will detect its absence in the templates for simulation; however, if the templates for guidance lack the content, observers will fail to detect its absence in the templates for simulation.^6^

Relatedly, Michel et al. (2024) suggest that patients with neglect fail to notice their visual deficits, which indicates a lack of awareness of the absence of visual experiences, due to failures in forming top-down expectations (templates for guidance) about normal vision. Similarly, Kim and Chong (2024b) demonstrated that individuals failed to detect degraded visual experiences (i.e., absence of high spatial frequency information) when visual inputs exceeded the capacity of cognitive access. Additionally, Kim and Chong (2024a) found that individuals failed to notice non-uniformities in their visual experiences around the visual field, such as greater degradation along the vertical than the horizontal meridians (horizontal–vertical anisotropy). These studies suggest that even healthy individuals often fail to form internal models (templates for guidance) of rich and detailed visual experiences throughout the visual field due to limited cognitive resources, which results in the lack of awareness of the absence of those experiences. Furthermore, previous studies revealed that individuals were more likely to detect degraded visual experiences (i.e., absence of color or detailed information) in the periphery when performing a task requiring peripheral information (i.e., extracting the gist of a scene) compared to a task requiring foveal information (Cho et al., 2025; Cohen et al., 2021; Cohen and Rubenstein, 2020). This flexibility in detecting degradation suggests that individuals can adjust their templates for guidance according to their specific goals.

Together, observers’ awareness (or lack thereof) of absence may reflect whether templates for guidance include specific representational contents for specific goal functions. This, in turn, provides insights into their models of the relationship between representational contents and functions, offering a window into their intuitions about consciousness.

## 7 Conclusion

Scientific studies of consciousness derive their legitimacy from individuals’ intuitions about consciousness. Therefore, if intuitions about consciousness are not sufficiently reliable or generalizable to the extent that consensus can be reached among individuals, scientific studies of consciousness will face significant challenges. Despite their importance, systematic investigations of the reliability and generalizability of intuitions about consciousness have not yet been actively conducted. Instead, existing studies often emphasize seemingly counterintuitive cases, suggesting that our intuitions about consciousness may be inaccurate and unreliable.

However, we propose that these counterintuitive cases may reflect inaccuracies not in our intuitions but in our measures of consciousness. When more refined measures are employed, partial awareness can provide intuitive explanations for these counterintuitive cases, which suggests that our intuitions about consciousness may be more accurate than previously assumed. Furthermore, by adopting the perspective that consciousness is a metacognitive model of internal representations, we reframe intuitions about consciousness as metacognitive models of the relationship between internal representations and functions. This functional reframing provides a rationale for why our intuitions about consciousness can be accurate and reliable. Our framework also aligns with recent calls to further extend and clarify the concept of meta-representation. For example, meta-representation may not only refer to a representation of a first-order representation, but also a representation of the relationship between first-order representations or between perception and action (Cleeremans et al., 2020; Kanai et al., 2025).

Additionally, reframed intuitions about consciousness can be systematically studied by connecting them to well-studied cognitive functions such as confidence, target templates, attention modes, and awareness of absence. Conversely, our framework emphasizes the necessity of explicitly considering the mechanisms through which internal representations and functions are linked to achieve a comprehensive understanding of the processes underlying these cognitive functions. It further suggests that these functions may be interrelated through shared mechanisms grounded in intuitions about consciousness.

Although the present study emphasizes the generality of intuitions about consciousness by focusing on extreme intuitive and counterintuitive cases, intuitions about consciousness can vary across individuals. This variability, however, can be accommodated within our framework, which proposes that intuitions about consciousness are optimized for effective and efficient information processing through experiences that differ across individuals. Accordingly, while our investigation begins by identifying commonalities across individuals, the ultimate goal is to explain and predict individual-level intuitions about consciousness by identifying the factors that systematically shape them.

In conclusion, we propose that establishing a reliable scientific foundation for intuitions about consciousness is not only necessary but also achievable for advancing scientific studies of consciousness.

## Data Availability

The original contributions presented in the study are included in the article/supplementary material, further inquiries can be directed to the corresponding author.

## Footnotes

1 One might argue that the partial awareness hypothesis is merely a post-hoc reinterpretation. However, the partial awareness hypothesis is empirically testable. For example, some of the studies cited in support of the partial awareness hypothesis are replication studies, including work on blindsight (Mazzi et al., 2016; Overgaard et al., 2008) and unconscious perception (Biderman and Mudrik, 2018; Gelbard-Sagiv et al., 2016; Lohse and Overgaard, 2019). In these studies, the hypothesis was falsifiable in the following sense: if previously reported dissociations between subjective and objective measures had been replicated using measures that explicitly account for partial awareness, the partial awareness hypothesis would have been challenged.

2 The stated aims of the functional reframing of intuitions about consciousness apply directly to the functional reframing of consciousness as a metacognitive model of internal representations. This functional reframing of consciousness is intended as a methodological and theoretical framework for the scientific study of consciousness, rather than as a reductive metaphysical claim. This framework offers systematic accounts of when and why consciousness arises and how it relates to other functionally well-defined phenomena. Moreover, it supports multi-level operationalization of consciousness across psychological, behavioral, neural, and computational measures.

3 Here, we distinguish between content and weight parameters, taking into account recent theoretical suggestions that the mechanisms underlying contents and the intensity of consciousness are dissociable (Fazekas and Overgaard, 2018; Morales, 2023). Further research is needed to clarify how the weight parameter can be integrated into these models.

4 This framework explains not only why intuitions about consciousness reliably track consciousness-function relations, but also why they sometimes fail to do so. Specifically, it proposes that intuitions about consciousness develop through experiences. On this view, such intuitions reliably track consciousness-function relations only when sufficient learning has occurred in contexts where those relations themselves are stable and reliable, such that strong intuitions about consciousness have been formed. In contrast, intuitions may be weak and fail to reliably track consciousness–function relations when they are applied to unfamiliar or novel contexts to which they do not readily generalize (Pearson, 2024).

5 Please note that this approach aims to identify a measure of consciousness that aligns with intuitions about consciousness, rather than to establish an objectively valid measure of consciousness. The latter falls outside the scope of our framework, as it focuses on functionally accessible aspects of consciousness and the possibility that phenomenal consciousness may be independent of observable functions cannot be tested within it.

6 Mazor and Fleming (2020) highlighted the distinction between awareness of absence (having an experience of no signal) and absence of awareness (having no experience of a signal). Their argument assumes that a second-order (metacognitive) inference about first-order (perceptual) representations is involved in the awareness of absence but not in the absence of awareness. This perspective aligns with our explanation that the awareness of absence does not arise directly from templates for simulation but instead requires further inferences about them, specifically through match decisions between templates for guidance and simulation. However, our explanation differs somewhat: While Mazor and Fleming emphasize the role of content-invariant higher-order states (Fleming, 2020), we underscore the importance of content-specific templates for guidance, as the awareness of absence inherently pertains to the absence of something specific. We propose that higher-order states indirectly influence the awareness of absence by constraining cognitive resources that can be allocated to templates for guidance.
